# Tumor-derived urinary exosomal long non-coding RNAs as diagnostic biomarkers for bladder cancer

**DOI:** 10.17179/excli2019-1683

**Published:** 2020-03-04

**Authors:** Maryam Abbastabar, Mohammad Sarfi, Abolfazl Golestani, Ali Karimi, Gholamreza Pourmand, Ehsan Khalili

**Affiliations:** 1 Department of Clinical Biochemistry, School of Medicine, Tehran University of Medical Sciences, Tehran, Iran; 2 Department of Urology, Shariati Hospital, Tehran University of Medical Sciences, Tehran, Iran; 3 Department of Urology, School of Medicine, Urology Research Center, Sina Hospital, Tehran University of Medical Sciences, Tehran, Iran

**Keywords:** LncRNA, exosome, bladder cancer, urine

## Abstract

Bladder cancer (BC) is the sixth most common malignancy in men and 17th in women. Exosomal long non-coding RNAs (lncRNAs) have been defined as a novel biomarker for BC. The aim of this study is to evaluate the clinical significance of urine exosomal PVT-1, ANRIL and PCAT-1 as a biomarker in BC patients with tumors classified as T1 or T2. Exosomes were isolated from urine of BC patients and healthy donors, then characterized according to their shape, size, and exosome markers by Electron Microscopy, Dynamic light scattering, and Western blotting. Exosomal lncRNAs extraction was done to determine the expression levels of PVT-1, ANRIL and PCAT-1 by qRT-PCR. ANRIL and PCAT-1 expression was significantly higher in BC patients compared to normal subjects. To evaluate the performance of the identified lncRNAs for BC detection, we performed ROC curves analysis. The diagnostic accuracy of ANRIL and PCAT-1, measured by AUC, was 0.7229 (sensitivity = 46.67 % and specificity = 87.5 %) and 0.7292 (sensitivity = 43.33 % and specificity = 87.5 %). Transcript levels of lncRNAs in urinary exosomes are potential diagnostic biomarkers in bladder cancer.

## Introduction

Bladder cancer (BC) is the sixth most common malignancy in men and 17th in women. In the United States, BC is the second most common genitourinary malignant disease, with 81,000 new cases and 17,000 deaths estimated in 2018 (Bray et al., 2018[4]). The incidence of BC correlates directly with age, so that 90 % of affected patients are older than 55 years (Kaufman et al., 2009[23]). Most BC occur in men and the number of cases is 3-4 times higher in men than that in women. There are numerous risk factors such as smoking, occupational exposures to aromatic amines and polycyclic aromatic hydrocarbons like aniline and benzo[a]pyrene, sex, aging, urinary tract infection , family history, arsenic in drinking water and unhealthy lifestyle, which can increase the possibility of developing BC (Burger et al., 2013[5]). The standard for BC diagnosis including cystoscopy, histopathological evaluation of biopsy samples and urine cytology which has several known disadvantages like false-positive and false-negative results, is uncomfortable and costly invasive (Kamat et al., 2016[22]; Kolodziej et al., 2016[25]). Additionally, current biomarkers, such as bladder tumor antigen (BTA) and nuclear matrix protein 22 (NMP22), unfits for low-grade disease due to the lack of diagnostic specificity and sensitivity (Yafi et al., 2015[42]; Leiblich, 2017[27]; Bhat and Ritch, 2019[3]). Therefore, it remains a great challenge to identify circulating non-invasive biomarkers and therapeutic targets for clinical management of BC patients and it’s urgent to find an effective biomarker to early diagnosis of BC. 

Eukaryotic lncRNAs are long, non-protein coding and single-stranded RNAs with more than 200 nucleotides in length that are closely related to the development and progression of human cancers (Esteller, 2011[14]). These molecules interact with DNA, proteins and other RNAs and in this way they are involved in various biological and pathological processes from transcription, translation, chromosomal dynamics, and telomere biology to subcellular structural organization (Hu et al., 2012[21]; Flynn and Chang, 2014[16]; Rossi and Antonangeli, 2014[41]; Fang and Fullwood, 2016[15]). LncRNAs can mediate epigenetic changes by recruiting chromatin remodeling complex such as Polycomb chromatin remodeling complex PRC1 and PRC2 to regulate the expression of neighboring protein-coding genes (Pandey et al., 2008[37]; Kotake et al., 2011[26]). Besides acting through PRC1 and PRC2, some lncRNAs act as competing endogenous RNAs and participate as a sponge for miRNA inhibition (Abbastabar et al., 2018[1]). In addition to the previously described mechanisms, lncRNAs can act in diverse strategies including they can be used as scaffold for proteins or whole protein complexes (Davidovich and Cech, 2015[12]) and modify the phosphorylation state of proteins via recruitment of phosphorylation motifs (Liu et al., 2015[29]). These characteristics have allowed lncRNAs to emerge as biomarkers in many types of cancer including BC. Recent observations suggest that extracellular vesicles like exosomes may act as transport vesicles for functional lncRNAs which may protect them from endogenous RNase activity in body fluid and result in a phenotypic effect within the recipient cell (Kogure et al., 2013[24]).

Exosomes (30-150 nm) are double-layer membrane vesicles of endocytic origin that are secreted from many cell types (Harding et al., 1983[18]). These vesicles are present in urine, serum, plasma, breast milk, saliva, and other bodily fluids and play a critical role in cell-cell communication via transmitting intracellular cargoes, such as protein, lipids, microRNA and lncRNAs (Nabet et al., 2017[34]). Recently, exosomal lncRNAs have gained attention, because abnormal expression of exosomal lncRNAs is considered a biomarker for their potential activity as biomarkers in several types of cancer such as BC. Antisense RNA In The INK4 Locus (ANRIL), Prostate Cancer Associated Transcript 1 (PCAT-1) and plasmacytoma variant translocation 1 (PVT-1) lncRNA act as oncogenic molecules in the cellular machinery (Prensner et al., 2014[38][39]; Chen et al., 2015[7]; Cui et al., 2016[10]). The previous study showed an overexpression of ANRIL, PCAT-1 and PVT-1 lncRNA in tissues samples of BC patients (Liu et al., 2015[29]; Zhu et al., 2015[49]; Liu and Zhang, 2017[32]). In this study, we aimed to evaluate the diagnostic utility of urinary exosomal ANRIL, PCAT-1 and PVT-1 lncRNA levels in BC patients in comparison whit healthy control. 

## Patients and Methods

### Study participants

A total 30 BC patients and 10 healthy control were obtained from the Shariati Hospital and Sina Hospital of Tehran City, Iran, from January 2018 to May 2018. All procedures were performed in accordance with the ethical standards. Informed consent was obtained from all individual participants included in the study. The BC individuals did not receive any chemotherapy before sample collection. BC patients were diagnosed by biopsy or histopathology, while the tumor was classified according to the tumor, node and metastasis (TNM) classification system of the International Union against Cancer. The mean age of BC patients was 55.84±11.96 years and 57.4±5.7 for healthy control.

All clinicopathological data for the BC samples, including age, sex, recurrence, tumor size, clinical T-category and histological grade, were obtained from the clinical and pathological records. The collected urine samples were centrifuged at 3000×g for 30 minutes at 4 °C to remove the cell debris. Subsequently, the pellet was aliquoted in to RNase‐free tubes and stored at −80 °C prior to further analysis.

### Urine exosome isolation

Norgen's Urine Exosome RNA Isolation Kit (Biotek Corporation, Thorold, ON, Canada) was used for isolation of urine exosomes by spin column method.

### Characterization of urine exosomes

Electron Microscopy (EM), Western blot analysis, and Dynamic light scattering (DLS), were used for assessment of size and morphology of the isolated exosomes.

#### Scanning electron microscopy (SEM)

For the SEM morphology investigation, after isolation of urinary exosomes using the Urine Exosome RNA Isolation Kit, Appendix B (Biotek Corporation, Thorold, ON, Canada), exosomes were dissolved in PBS and visualized under scanning electron microscopy (QUANTA SEM system; FEI Company, Hillsboro, OR, USA).

#### Transmission electron microscopy (TEM)

For the TEM morphology investigation, 10 ml of exosomes pellet were placed on formvar carbon-coated 200-mesh copper electron microscopy grids, and then grids were stained with 2 % uranyl acetate for 1 min at room temperature. Finally, exosomes were imaged under transmission electron microscopy (Zeiss, EM10C, 100kv, Germany).

#### Western blotting

Total protein of urinary exosomes was extracted with Urine Exosome RNA Isolation Kit, Appendix A (Biotek Corporation, Thorold, ON, Canada) according to manufacturer's instructions. Equal amounts of protein lysates were subjected to Western blotting analysis by using anti-CD63 antibody (SantaCruz Biotechnology, Dallas, TX, USA), according to standard protocols.

#### Dynamic light scattering assessments

The size distribution of exosomes was determined using Zetasizer Nano ZS (Malvern Instruments, Malvern, UK) based on the company guidelines.

### Exosomal RNA isolation

Total exosomal RNA was isolated from the urine samples using the Urine Exosome RNA Isolation Kit (Biotek Corporation, Thorold, ON, Canada) following the manufacturer's instructions. The purity of isolated RNA was evaluated by OD260/280 using NanoDrop spectrophotometer (Thermo Fisher Scientific).

### Quantitative real-time PCR analysis

Purified RNA was reversely transcribed into cDNA using RevertAid First Strand cDNA Synthesis (Takara, Tokyo, Japan). qRT-PCR was performed using SYBR Premix Ex Taq II (Takara, Dalian, Liaoning, China) under the following conditions: 94 °C for 5 minutes, 40 cycles of 94 °C for 1 minutes, 58 °C for 1 minutes, and 72 °C for 1 minutes. All experiments were formed in duplicate in 20 μL total volumes. 5s rRNA was used for normalization and the relative expressions of target genes were calculated by comparative cycle threshold (Ct) (2^−ΔΔCt^) method. The nucleotide sequence of primers used in expression analyses are shown in Table 1(Tab. 1).

### Statistical methods

All statistical analyses were performed using SPSS v18.0 (SPSS Inc., Chicago, IL, USA). Shapiro-Wilk test was applied to determine if the data follows a normal distribution. The differences in the expressions of lncRNAs between BC patients and healthy controls were assessed by non-parametric Mann-Whitney U test. For all assays *p *< 0.05 was considered statistically significant.

### Ethical approval

This study was reviewed and approved by the Ethics Committee of Tehran University of Medical Science, and all of the participants signed an informed consent form.

## Results

### General data of patients

All tumor specimens were classified and graded by pathologist. Histological grading was performed using the 1973 WHO and the 2004 WHO/International Society of Urological Pathology classifications (Lopez-Beltran and Montironi, 2004[33]). The T-category was assessed according to the 2002 American Joint Committee on Cancer staging system (Greene and Sobin, 2008[17]). The study participants, clinical information of age, recurrence, grade, T-category and tumor size are summarized in Table 2(Tab. 2).

### Isolation and characterization of exosome

**Figure 1:**
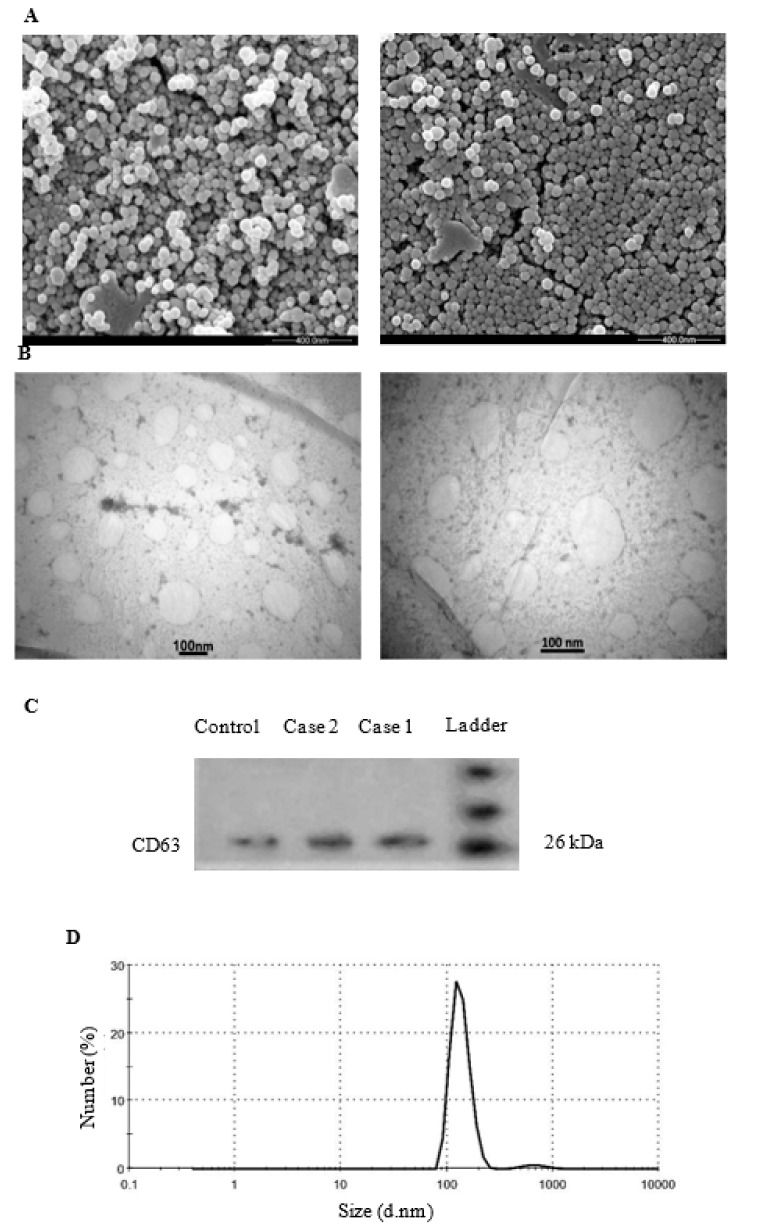
Characterization of urinary exosomes. A) Scanning electron micrographs of exosomes isolated from urine samples of BC patients (left) and control (right), which exhibited a cup-shaped membrane morphology with a diameter of 60-130 nm. The size of exosomes was not different between study groups (scale bar = 400 nm) B) Transmission electron microscopy images of isolated exosomes from urine samples of BC patients (left) and control (right), (scale bar = 100 nm). C) Urinary exosomes-enriched protein marker CD63 (26kDa) was analyzed by Western blotting in exosomes and D) The sizes of urine exosomes were characterized via DLS and the majority of vesicle particles were mainly between 60 and 190 nm in diameter.

The morphology and phenotype of isolated particles from urine were identified by EM, Western blot analysis, and DLS. First, the morphology and size range of the exosomes isolated from BC patients and healthy controls was observed directly through SEM and TEM. The result demonstrated that urinary exosomes have diameter of 70-150 nm, the expected exosome size range (Figure 1A and 1B(Fig. 1)). Secondly, we evaluated the presence of exosome marker CD63 in the vesicle isolated from the urine of BC patients and healthy controls via Western blot analysis. We found it expressed in our samples (Figure 1C(Fig. 1)). Finally, the results were also confirmed by DLS assessment, which showed that isolated vesicles displayed diameter ranging between 60 and 190 nm (Figure 1D(Fig. 1)). In conclusion, the above properties analysis indicated that urine derived particles collected in our experiment were identified as exosome.

### Expression of ANRIL, PCAT-1 AND PVT-1 genes in urinary exosomes

**Figure 2:**
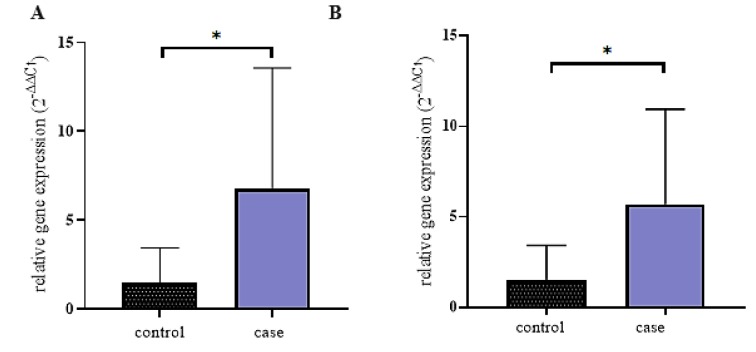
ANRIL and PCAT-1 in exosomes. Expression levels of serum exosomal ANRIL (A) and PCAT-1 (B) in healthy controls and BC patients were determined by qRT-PCR. 5s rRNA was used as an endogenous control. All qRT-PCR reactions were performed duplicate, and values are presented as mean ± SD. Since the data were non-normally distributed, Mann-Whitney test was performed; *n *= 40 [10 healthy controls, 30 BC patients].

We performed qRT-PCR to assess the presence of ANRIL, PCAT-1, and PVT-1 in exosomes isolated from urine of BC patients and healthy control. We detected ANRIL (Figure 2A(Fig. 2)) and PCAT-1 (Figure 2B(Fig. 2)) in exosomes from BC patients and healthy controls. The expression levels of ANRIL and PCAT-1 were normalized using 5s rRNA because our CT values show that this RNA is stably expressed in all of our samples. RT-qPCR results showed that ANRIL and PCAT-1 were significantly (*p*<0.05) higher in the urinary exosomes of BC patients compared with healthy controls. However, PVT-1 is not detected in urinary exosomes of BC patients (Data not shown).

### Associations between the urinary exosomes-derived lncRNAs and clinicopathological features

We tested the relationship between exosomal ANRIL and PCAT-1 levels and clinicopathological features in BC patients. As shown in Table 3(Tab. 3), there was no clear relationship between exosomal ANRIL and PCAT-1 levels and clinicopathological features including recurrence, grade, T-category and tumor size. 

### Validation of the diagnosis performance of the lncRNA panel

**Figure 3:**
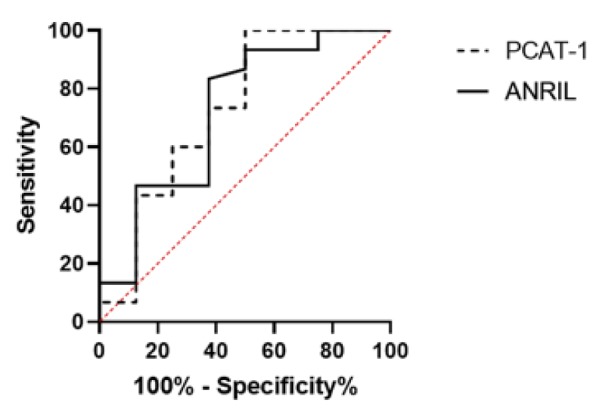
The results of ROC curve analysis for the diagnostic value of ANRIL and PCAT-1 in the urinary exosomes of BC patients, the area under the curve (AUC) 0.7229 and 0.7292 for ANRIL and PCAT-1, respectively.

To evaluate the performance of the identified lncRNAs for BC detection, we performed ROC curves analysis. The diagnostic accuracy of ANRIL and PCAT-1, measured by AUC, was 0.7229 (95 % CI = 0.4981 to 0.9477, sensitivity = 46.67 % and specificity = 87.5 %) and 0.7292 (95 % CI = 0.4949 to 0.9634, sensitivity = 43.33 % and specificity = 87.5 %), respectively. Based on the calculated AUC values, ANRIL and PCAT-1 transcript levels had the fair performance in differentiation of BC from total controls (Figure 3(Fig. 3)). 

For more results see the Supplementary data.

## Discussion

Early diagnosis of cancer will play an important role in the successful treatment of patients. Biomarkers play an important role in the clinical management of patients in oncology, including estimating risk of disease, screening, diagnosis, prognosis and predicting drug efficacy or responses to various treatments (Hayes et al., 2014[19]). Cancer biomarkers cover a broad range of biochemical entities, such as include tumor-derived circulating tumor cells, circulating tumor DNA, proteins, small metabolites, phosphoproteins and exosomes (Palmirotta et al., 2018[36]). Exosomes are nanosized membrane vesicles with an endosomal origin that are secreted in to body fluids (Harding et al., 1983[18]). Emerging evidence indicates that exosomes contain the characteristic proteins which belong to their biogenesis process, including CD63, CD81, Alix and Tsg101 that have been used as exosome markers. Exosomes also carry mRNA, microRNA and lncRNA molecules (Nabet et al., 2017[34]). Several studies have shown that proteins, miRNAs and lncRNAs contained in exosomes might be potential biomarkers of different cancers (Chen et al., 2014[6]; Belov et al., 2016[2]). 

LncRNAs as non-coding RNAs longer than 200 nucleutide in length, can be found in different biofluids like plasma, serum and urine, in which they can be transported and protected from RNase activity through lipoprotein binding and exosome packaging (Dragomir et al., 2018[13]). Recently, many BC-associated lncRNAs have been identified. Zhang et al. reported that dysregulated colon cancer-associated transcript 1 (CCAT1) is required for invasion, migration and proliferation in bladder urothelial carcinoma cell lines, which may provide an efficient classification tool for clinical prognosis evaluation of BC (Zhang et al., 2019[48]). Metastasis Associated Lung Adenocarcinoma Transcript 1 (MALAT-1), PCAT-1 and SPRY4 Intronic Transcript 1 (SPRY4-IT1), and other lncRNAs such as PVT-1 have been reported to be associated with BC development (Cui et al., 2017[11]; Zhan et al., 2018[46]).

In the present study, we evaluated expressions of three lncRNAs ANRIL, PCAT-1 and PVT-1 in urinary exosomes of BC patients and normal subjects. Of note, we found significant difference in the expression of two lncRNAs including ANRIL and PCAT-1 between BC patients and normal samples. Several studies have been shown that up-regulation of ANRIL is regarded as a risk factor in several types of human cancers, including gastric, cancer, lung cancer, breast cancer, esophageal squamous cell carcinoma, hepatocellular carcinoma etc. (Zhang et al., 2014[48]; Chen et al., 2015[7]; Lin et al., 2015[28]; Nie et al., 2015[35]). Several study reported that the tissue levels of ANRIL were higher versus the corresponding adjacent non-tumor tissues, which implied that ANRIL might play an oncogenic role in BC (Hoffmann et al., 2015[20]; Zhu et al., 2015[49]). In the present study, we found that ANRIL expression was significantly higher in BC patients compared to controls.

PCAT-1 is another cancer associated lncRNA dysregulated in many malignant tumors (Liu et al., 2015[31]). This lncRNA is located in the chromosome 8q24 gene desert and it has diverse biological effects, including invasion, metastasis, apoptosis, cell proliferation and cell cycle by interacting with different types of molecules like Breast cancer type 2 (BRCA2) tumor suppressor gene and c-Myc protein (Prensner et al., 2014[38][39]). Several previous studies have pointed to the role of PCAT-1 in the pathogenesis of human BC. PCAT-1 was found to be upregulated in BC compared to paired normal urothelium. Cell proliferation inhibition and apoptosis induction were also observed in PCAT-1 small hairpin RNA (shRNA)-transfected BC T24 and 5637 cells (Liu et al., 2015[31]).

PVT-1, which was first discovered as an activator of MYC in murine plasmacytoma variant translocations, is located at 8q24.21 (Cory et al., 1985[8]). This lncRNA is abundant in several tumor types, including lung, gastric, and prostate cancers (Cui et al., 2016[9]; Liu et al., 2016[30]; Yuan et al., 2016[45]) and plays oncogenic role via three different mechanisms: interacting with MYC, participating in DNA rearrangements and encoding microRNAs (Cui et al., 2016[10]). Despite the established oncogenic role of PVT-1 in BC (Cui et al., 2017[11]), we could not detect it in urinary exosome of BC patients. 

Despite significant efforts in utility exosomes for disease detection and treatment, progress has been aggravated by challenges. One of the controversial issues in exosomal studies is their extraction from biological fluids (Ramirez et al., 2018[40]). There are several EV isolation and characterization techniques available such as differential centrifugation, size exclusion chromatography and using a total exosomal RNA and protein isolation kit (Yakimchuk, 2015[43]). For the isolation of urine exosomes, we used Norgen's Urine Exosome RNA Isolation Kit by column-based system, which has been successfully used to extract exosomes (Yazarlou et al., 2018[44]). Using this method, we successfully isolated exosomes from the urine of BC patients and healthy donors.

This study is one of the few reports that analyze expression levels of lncRNAs in exosomes isolated from urine of BC patients. Transcript levels of ANRIL and PCAT-1 in urinary exosomes are potential diagnostic biomarkers in bladder cancer.

## Notes

Maryam Abbastabar and Mohammad Sarfi contributed equally as first authors.

## Conflict of interest

The authors declare no conflict of interest.

## Acknowledgements

This work was financially supported by grant (96-04-30-36959) from the Deputy of Research, Tehran University of Medical Sciences.

## Supplementary Material

Supplementary data

## Figures and Tables

**Table 1:**
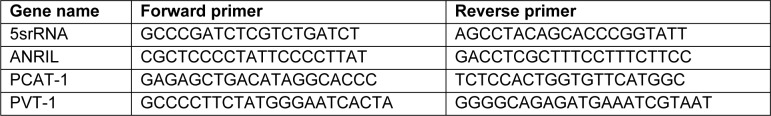
Nucleotide sequence of primers used for expression analysis of lncRNAs in urinary exosomes

**Table 2:**
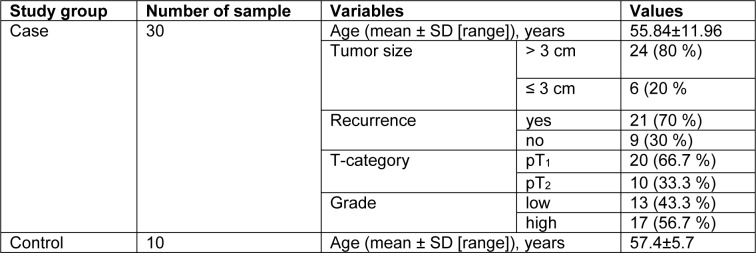
Clinical characteristics of BC patients and healthy control

**Table 3:**
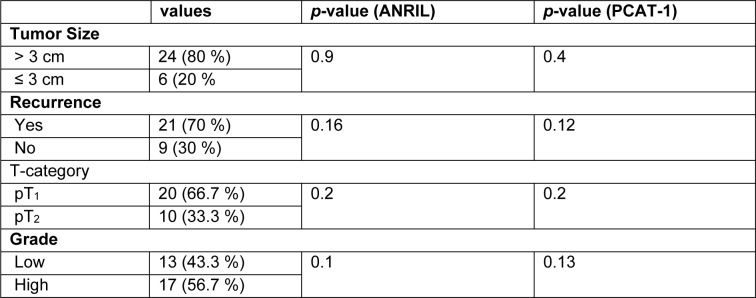
Associations between relative expression of lncRNAs in urinary exosomes of TCC patients and clinicopathological data
